# New Male Specific Markers for Hop and Application in Breeding Program

**DOI:** 10.1038/s41598-019-50400-z

**Published:** 2019-10-02

**Authors:** Andreja Čerenak, Zala Kolenc, Petra Sehur, Simon P. Whittock, Anthony Koutoulis, Ron Beatson, Emily Buck, Branka Javornik, Suzana Škof, Jernej Jakše

**Affiliations:** 1 Slovenian Institute of Hop Research and Brewing, Cesta Žalskega tabora 2, 3310 Žalec, Slovenia; 2 Hop Products Australia, 446 Elizabeth Street Hobart, Tasmania & School of Natural Sciences, University of Tasmania, Private Bag 55, Hobart, Tasmania Australia; 3 University of Tasmania, School of Natural Sciences, Private Bag 55, Hobart, TAS 7001 Australia; 4 The New Zealand Institute for Plant & Food Research Limited, Palmerston North Research Centre, Private Bag 11600, Palmerston North, 4442 New Zealand; 5 University of Ljubljana, Biotechnical Faculty, Agronomy Department, Jamnikarjeva 101, 1000 Ljubljana, Slovenia

**Keywords:** Biotechnology, Plant sciences

## Abstract

Male specific DNA sequences were selected from a Diversity Arrays Technology (DArT) mapping study to evaluate their suitability for determination of the sex phenotype among young seedlings in a hop (*Humulus lupulus* L.) breeding program. Ten male specific DArT markers showed complete linkage with male sex phenotype in three crossing families. Following optimization, four were successfully converted into PCR markers and a multiplex PCR approach for their use was developed. Among 197 plants (97 from the world collection; 100 from three segregating families), 94–100% positive correlation with sex phenotypic data was achieved for the single PCR amplification, whereas the multiplex approach showed 100% correlation. To develop a fast and low-cost method, crude sample multiplex PCR was evaluated in 253 progenies from 14 segregating populations without losing accuracy. The study describes, for the first time, the routine application of molecular markers linked to male sex in an intensive Slovenian hop breeding program. The methods described could be employed for screening of sex at the seedling stage in other hop programs worldwide, thereby saving resources for desirable female plants.

## Introduction

Hop (*Humulus lupulus*) is a perennial, dioecious, wind-pollinated species. The lateral shoots of the female hop plant carry the cones that are essential to the beer brewing industry. Plant secondary metabolites that accumulate in hop cones contribute to bitterness (bitter acids), aroma (essential oils), and stability in beer (polyphenols^1,2^), and exhibit pharmacological effects (anti-carcinogenic, anti-inflammatory and phytoestrogenic^3^). Global hop production occupies up to 60.000 ha^4^, including acreage in Europe, USA, China, South Africa, Australia and New Zealand. Hop cultivars are clonally propagated for deployment, and as such may be highly locally adapted, with the result that each hop growing jurisdiction tends to support a local hop breeding and cultivar development program.

Hop breeding programs typically aim to increase yield (through plant architecture or resistence/tolerance to abiotic or biotic stresses), and improve quality. Progress in hop breeding is limited by the biology of hop. Hop can take up to two years under field conditions to reach reproductive maturity^5,6^. Furthermore, as the female hop cones are the commercial product it is difficult to relate the commercial requirements with the male phenotype since cones do not develop on male plants. These factors, combined with high levels of heterozygosity, limit progress in hop breeding. Furthermore, the presence of male plants adjacent to commercial hop fields or breeding populations results in the presence of seed in hop cones, which increases the weight of cones, but can reduce their brewing quality, and therefore their value. Since the hop plant is wind pollinated, a single male plant in the hop field or its vicinity can cause broad scale damage to the crop^7^.

In dioecious species such as hop, each individual normally produces either male or female reproductive organs^8^. Dioecy has been observed in about 9% of the ~319.000 known plant species, with levels higher among bryophytes (62%) and gymnosperms (36%), while in angiosperms only about 5% of species are dioecious^9^. In almost all studied dioecious species, the sex phenotype is linked to genetic differences between male and female plants at least at one locus on a pair of chromosomes^10^. Renner and Ricklef^11^ also reported that dioecy is more common in wind-pollinated species in comparison to animal pollinated plants.

Molecular markers are a useful tool for determination of sex in dioecious plants before the phenotypic differences become detectable as the plants enter their reproductive phase. Several reports on identification of sex-specific markers in other plants have been published, such as in rattan *Calamus guruba* Buch.-Ham^12^, *Momordica dioica*^13^, *Coccinia grandis*^14^, *Carica papaya*^15^, and *Cannabis sativa*^16^.

Hop (*H. lupulus* var. *lupulus*) is normally diploid (2n = 2x = 20) with nine autosomal bivalents and two heteromorphic sex chromosomes. Female plants have a pair of X chromosomes, while male plants have an XY pair with the Y chromosome being smaller^8,17–19^. In all other species of the genus *Humulus*, such as *H. lupulus* var. *cordifolius*, *H. japonicus*, *H. yunnanensis* and *H. acetosa*, the sex is expressed based on a multi-sex chromosome system^20–22^. Expression of sex phenotype in hop is also influenced by the X: autosomes ratio^8^, indicating that the genes controlling pollen development are in the sex determining region and structural genes located on autosomes^17,21^. In hop, monoecious plants occasionally occur, especially among progeny of specific crosses. Škof *et al*.^23^ revealed that a high percentage of monoecious hop plants were triploids. Experimental work of this study focusses on diploid germplasm.

Since the hop karyotype contains sex chromosome Y, which is transmitted from generation to generation only by the male line, it was first assumed that majority of polymorphisms on Y chromosome should be male specific. Polley *et al*.^6^, on the basis of one cross, isolated a male molecular marker linked to sequences on the Y chromosome. Čerenak and Javornik^24^ tested the published marker in the Slovenian breeding program, but the marker did not multiply in diploid and tetraploid forms of the Japanese male hop No3-38, and therefore, there was an assumption that the marker developed was specific to European hops. Patzak *et al*.^5^ further tested its suitability in four families from the Czech breeding program and they also observed that the marker was not absolutely linked to the male phenotype. Seefelder *et al*.^25^ were the first to construct a genetic linkage map of the male hop, and they found 24 molecular markers which were sex specific. Danilova and Karlov^26^ were successful in the development of male specific molecular markers with the inter-simple sequence repeat polymerase chain reaction (ISSR-PCR) method on hop plants of Russian and European origin. Čerenak *et al*.^27^ published a genetic linkage map where simple-sequence repeat (SSR) marker HlAGA7 mapped to the male locus. Jakše *et al*.^28^ further described the HlAGA7 marker which appeared to distinguish male, female and monoecious plants in two Slovenian populations. In the study of McAdam *et al*.^29^ the marker HlAGA7 showed complete linkage to the male sex phenotype in a New Zealand mapping population. Even though the marker proved to be perfectly associated with the male sex, the use of this particular marker was limited due to the technical difficulties of using SSR with multiple alleles. Buck *et al*.^30^ developed four sex linked markers; three sequenced characterised amplified region (SCAR) markers, and one high-resolution melting (HRM) curve analysis marker the latter of which has been successfully applied within the New Zealand hop breeding programme^31^. Recently, Hill *et al*.^22^ identified a pseudoautosomal region (PAR), and male-specific regions of the Y-chromosome, along with genes located in these parts of the sex chromosomes.

In order to develop an efficient method to determine the sex phenotype among young seedlings in a hop breeding program, we developed a multiplex PCR approach amplifying four male specific markers and a chloroplast specific DNA fragment. Male specific markers were idenitified from a DArT (Diversity Arrays Technology^32^ genetic mapping study^33^. Selected DArT markers and the developed multiplex PCR approach were validated across a broad spectrum of hop genetic resources, in progeny of specific crosses, and for routine application in the Slovene breeding program. Furthermore, we evaluated these markers on crude sap samples to avoid the tedious step of DNA isolation, with promising results.

## Materials and Methods

### Phenotypic determination of sex in hop plants

Sex phenotype was determined by in-field visual observation of seedlings during the flowering phase (from June to July) in years 2014 and 2015, on 1 and 2 year old plants (respectively). The results of phenotypic sex determination were compared against the molecular markers.

### DNA extraction

Total genomic DNA was extracted from fresh plant material (leaves, plant buds, and tissue cultures) according to the modified CTAB protocol^34^. DNA concentration was quantified by means of fluorometry and samples stored at −20 °C.

### Male specific DArT marker discovery

#### Plant material

Three mapping populations from: (i) New Zealand (‘Nugget’ × ‘SBL3/3’, 170 plants^30^); (ii) Slovenia (‘Hallertauer Magnum’ × ‘SBL2/1’, 92 plants^27,29^); and (iii) USA (‘Perle’ × ‘USDA19058M’, 124 plants^35^) were included in sex linked marker development.

#### DArT male linked markers

In the framework of the hop DArT consortium mapping project, all markers were sequenced by Sanger technology. The genotyping data for the Slovenian population were searched for DArT markers showing complete linkage with male sex phenotype, while being absent in female plants (i.e., no recombination between the DArT marker and trait of interest). The resulting set of markers was compared against the phenotypic data from the other two mapping populations, to infer male plants and to identify any additional male sex-linked markers.

Identified male linked sequences were edited and assembled in CodonCode Aligner (ver. 7.1.2) and submitted to GenBank (MG744425-MG744432). A hop chloroplast specific sequence named contig18 (GenBank MG744433) was obtained from our recent hop transcriptome project^36^.

#### Primer construction

Primer pairs for use in a single PCR were constructed using PRIMER3^37^ web version using the default program parameters. Primers for the multiplex approach were designed using the MPprimer tool^38^ specially developed to account for multiplex conditions, using default program options. Developed primers are presented in Tables 1 and 2.

**Table 1:** Primers for amplifying male sex linked DArT single markers.

DArT marker	Primer sequence for (5′-3′)	Primer sequence rev (5′-3′)	Expected amplicon length (bp)
hPb-361327, hPb-363461, hPb-718886 - > hPb-CONT	AGGGTGTGCAAGGTTGAAAT	CTCCAATCGAACCTCGTGAT	444
hPb-365890	TTCCTTTCCGTGGACCTATG	GGTTCTACCCCTTGGCCTAC	203
hPb-366371*	TGGAATGTCTGTTGCTTTGC	GTCACCCAGCGATTGTCTTT	252
hPb-716926*	TCAAAAGCTGGCAAAACACA	TTTTACTGGCATGACTACAACAGA	277
hPb-718821	AAAGCTCCACAAAGCTGCAT	TGCCCATAGTTGCTCACTTG	266
hPb-719005	TTGGATGATGGAAGATGCTG	TTTCTCGCCCTTAACATTCG	221

^*^For these two markers amplification was not achieved.

**Table 2:** Male specific, and chloroplast DNA derived positive control primer pairs set for multiplex PCR test.

Marker	Primer pair sequence (5′-3′)	Primer pair sequence (5′-3′)	Amplicon length (bp)
contig18	TCCTGGTCCCTGCGGAAAGGAA	AGAGCGCGCCCCTGATAATTGC	569
hPb-718821	TCGATAGCTGCATCAGCCTGAGA	TCCCACTGGCCTTTTGGCCTCT	455
hPb-719005	CCGACACCGGTCAAAGCCAAGA	TGGCATGGACATACTAAATCCAGCATC	366
hPb-365890	AGTGCTGGAGCAAGACACCCCT	TGTCGCCCATAGGTCCACGGAA	229
hPb-CONT	TCATCAGCAGGTGGGTCAGGCA	TCCGCACTTCTCTCACAGGCGA	124

### Single PCR marker reaction

#### Plant material

203 different genotypes (including cultivars, wild female and male plants, and monoecious plants; Supplementary Information 1) and 100 breeding lines (Table 3) with known sex phenotypes from three different families were used in PCR amplification.

**Table 3:** Comparison of single PCR amplification with two loci (hPb-CONT, hPb-719005) and multiplex PCR in three different crossing families (crossing year 2011).

Crossing family	No. of hop plants	No. of male plants^a^	No. of female plants^a^	Correctness of sex prediction by primer hPb-CONT (%)	Correctness of sex prediction by primer hPb-719005 (%)	Correctness of sex prediction by multiplex PCR (%)
‘Hallertauer Tradition’ × 300/166	32	8	24	94	94	100
‘Taurus’ × 305/27	20	4	16	95	100	100
20/27 × 284/113	48	10	38	96	96	100
**Total/average**	100	22	78	95	96	100

^a^Phenotypic sex determination.

#### PCR amplification

The amplification was performed in 20 μl solution containing 1x PCR buffer, 2 mM MgCl_2_, 0.2 mM each dNTPs, 0.5 μM primers, 0.5 U of *Taq* DNA polymerase and 20 ng of genomic hop DNA using the following thermal cycling protocols: 1) primers hPb-CONT: 94 °C for 5 min, followed by 40 cycles of 30 sec at 94 °C, 30 sec at 58 °C and 90 sec at 72 °C, 2) primers hPb-719005 (touchdown protocol): 94 °C for 5 min, followed by 10 cycles of 30 sec at 94 °C, 30 sec at 62 °C (decreased -1 °C each cycle), 1 min at 72 °C and 30 cycles at 94 °C for 30 sec, 52 °C for 30 sec and 72 °C for 1 min, 3) primers hPb-365890 and hPb-718821: 94 °C for 5 min, followed by 40 cycles of 30 sec at 94 °C, 30 sec at 67 °C and 1 min at 72 °C. All reactions were completed by incubating at 72 °C for 8 min.

Only two primers, hPb-CONT and hPb-719005 (Table 1) were further used for amplification across three crossing families (Table 3). Amplified PCR products were separated on 2% agarose gel and visualized by ethidium bromide staining.

### Multiplex PCR marker development

#### Plant material

A total of 97 hop accessions of different origins (Supplementary Information 1) were included in the optimization of multiplex PCR reaction. The sample set comprised of 24 male and 73 female genotypes. Additional samples from three families (Table 3) were used for comparison of the results obtained from single and multiplex PCR reactions.

#### Multiplex PCR amplification

Single primer-pair PCRs were carried out initially, for each of the five multiplex primer pairs listed in Table 2. After initial confirmation by single pair amplification, the multiplex amplification was optimized by varying the primer concentration. Optimized PCR conditions in 15 μl reactions were as follows: 40 ng DNA, 1x QIAGEN Multiplex PCR Master Mix, 1x Q-Solution and primers at following concentrations: 0.2 μM for primers hPb-CONT, hPb-365890 and hPb-719005, 0.4 μM for primer hPb-718821 and 0.04 μM for primer contig18. Different primer concentrations are crucial to achieving a comparable rate of amplification of five different fragments. For example, contig18 which is of chloroplast origin requires much lower primer concentration, due to higher number of copies present in nucleic acid extract compared to the nuclear DNA. Amplification was carried out using the following thermal cycling touchdown protocol: 95 °C for 15 min, followed by 8 cycles of 30 sec at 94 °C, 90 sec at 65 °C (decreased 1 °C each subsequent cycle) and 90 sec at 72 °C. The amplification continued for 27 cycles at 94 °C for 30 sec, 57 °C for 90 sec and 72 °C for 90 sec. The reactions were completed by incubation at 72 °C for 10 min. PCR products were separated on 2% agarose gel and visualized by ethidium bromide staining.

### Crude sample multiplex PCR amplification

#### Plant material

As a first optimisation step, 10 samples of female varieties and 10 male plants were used for amplification of crude sample PCR. Afterwards, 253 hop plants at the seedling stage from Slovenian hop breeding program representing 14 crossing families were analysed by using crude sample multiplex PCR amplification (Table 4).

**Table 4:** Crude sample multiplex PCR amplification in 14 different crossing families (crossing year 2013).

Crossing family	No. of plants	No. of male plants (phenotypic determination)	No. of female plants (phenotypic determination)	Correctness of sex prediction by crude sample multiplex PCR (%)
‘Aurora’ × 173/132	11	2	9	100
‘Saazer’ × 305/27	18	4	14	100
‘Aurora’ × 21340	34	11	23	100
‘Buket’ × 2/1	26	9	17	100
‘Columbus’ × 25/234	29	0	29	100
‘Columbus’ × 24/80	13	0	13	100
‘Dana’ × 305/27	24	0	24	100
‘Dana’ × 141/109	19	1	18	100
‘Bobek’ × 85/169	20	1	19	100
‘Hallertauer Tradition’ × 300/166	10	1	9	100
‘Hallertauer Tradition’ × 162/75	29	1	28	100
‘Hallertauer Tradition’ × 159/106	5	0	5	100
‘Chinook’ × 310/48	8	1	7	100
‘Cascade’ × 25/234	7	7	0	100
Sum	253	38	215	100

#### Crude Sample multiplex PCR amplification

Crude sample multiplex PCR amplification was developed using Kapa 3 G Plant PCR kit (Kapa Biosystems) utilizing fast extraction of crude DNA extract. Leaf disc circles (1 cm diameter) were excised by a puncture tool and immersed in 200 μl of extraction buffer (0.5 M Tris-HCl, 1 mM EDTA (pH = 8.0), 2% β-mercaptoethanol) with two steel beads (5 mm). Tissue was homogenized in TissueLyser (Qiagen) with 10 rotations per second for 30 sec. Samples were heated at 95 °C for 5 min, cooled (-20 °C) for 10 min and centrifuged at 12,000 g for 10 min. Supernatant of crude extract was diluted in ratio 1:9 in sterile dH_2_O. Two μl of diluted crude DNA extract was used in 10 μl PCR solution containing 1x PCR buffer, 1.25 mM MgCl_2_, Enhancer (diluted 1:50), 0.2 U of KAPA3G Plant DNA polymerase and primers in same concentration as determined previously. The PCR amplification profiles and agarose gel electrophoresis analysis were the same as described for multiplex reactions (see Multiplex PCR amplification).

## Results

### DArT male linked markers discovery

In the Slovenian mapping family (‘Hallertauer Magnum’ × ‘SBL2/1’) represented by 92 plants, 9 were phenotypically male. Based on this observation, 10 DArT markers (hPb-361327, hPb-363461, hPb-365890, hPb-366371, hPb-715987, hPb-716314, hPb-718821, hPb-718886, hPb-719005, and hPb-716926) were discovered that were present in the male parent, absent in the female parent and present in all male siblings. Based on this information these markers were searched in the New Zealand (‘Nugget’ × ‘SBL3/3’) and USA (‘Perle’ × ‘USDA19058M’) mapping populations for being present in male parent and in male siblings. Three markers (hPb-365890, hPb-716314 and hPb-719005) were confirmed to be present in all three families, 5 were common between Slovenian and New Zealand’s families (hPb-718886, hPb-718821, hPb-715987, hPb-363461, and hPb-361327), while one marker was unique to each of the Slovenian family (hPb-716926) and USA family (hPb-366371). Therefore, there were a total of 10 DArT markers specific to the male phenotype among those three families (Table 5).

**Table 5:** Presence of male linked DArT markers in three different mapping families^29^.

DArT marker	Accession number	Mapping family from^29^		
		'Hallertauer Magnum’ × 2/1	'Nugget’ × 3/3	'Perle’ × 19058M
hPb-361327*	MG744425	X	X	—
hPb-363461*	MG744426	X	X	—
hPb-365890	MG744427	X	X	X
hPb-366371	MG744428	—	—	X
hPb-715987	/	X	X	—
hPb-716314	/	X	X	X
hPb-716926	MG744429	X	—	—
hPb-718821	MG744430	X	X	—
hPb-718886*	MG744431	X	X	—
hPb-719005	MG744432	X	X	X

^*^DArT markers with almost identical sequences (sequence comparison in Supplementary Information 2).

For two DArT markers, hPb-715987 and hPb-716314, quality DNA sequences were not obtained, and therefore they were omitted from analysis. Comparison of the remaining eight male linked DArT marker sequences revealed that three markers hPb-361327, hPb-363461 and hPb-718886, are almost identical showing three A- > G transitions and probably representing two alleles (Supplementary Information 2: Alignment of sequences). The primer pair developed based on their alignment was named hPb-CONT; the other primers retained the DArT marker nomenclature. Together, six unique, male linked sequences were further tested in single PCR amplification.

### Initial single PCR screening of male linked markers

Single PCR primers were developed (Table 1) and PCR conditions optimized on a set of four female and four male hops comprising cultivars ‘Magnum’, ‘Perle’, ‘Comet’, and ‘Fuggle’ and male breeding lines 2/1, 3/3, 19058, and 29-70-54 for four DArT markers, including the contiguous sequence hPb-CONT (representing hPb-361327, hPb-363461, hPb-718886), hPb-365890, hPb-718821 and hPb-719005. These four male-specific markers were further screened in 122 female genotypes (117 cultivars and 5 wild hops), 44 male genotypes and 37 monoecious genotypes (20 predominantly male phenotype – Mf; 16 predominantly female phenotype – Fm and 1 plant in which neither male nor female flowers clearly predominate - FM - Supplementary Information 1). In summary, no male specific marker was successfully amplified in female genotypes, while the success of identifying males varied. For example, markers hPb-CONT and hPb-365890 each failed to amplify in two male genotypes (hPb-CONT: No3-38 and 284/113; hPb-365890: 19058 and 120/13). The marker hPb-718821 was not amplified in one male genotype (19058), and hPb-719005 did not amplify in four males (No3-38, 85/169, 19058 and 120/13). Interestingly, male specific markers were amplified in Mf hermaphroditic plants while not in Fm plants. For two DArT marker sequences (hPb-366371 and hPb-716926) amplification could not be achieved without varying the PCR conditions.

**Figure 1:**
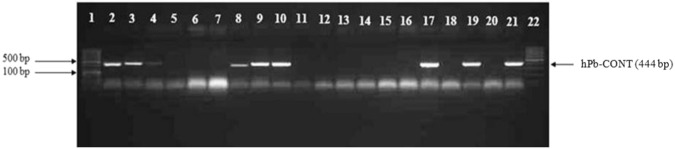
PCR amplification of male specific marker hPb-CONT in 20 hop plants from crossing family ‘Aurora’ × 305/28. Samples in lane 2–4, 8–10, 17, 19, 21 are male hop plants. Samples in lane 5–7, 11–16, 18, and 20 are female hop plants. Lane 20 and 21 are the female parent ‘Aurora’ and the male parent 305/28 respectively. Lane 1 and 22 contained a 100 bp size marker. Full-length gel is presented in Supplementary Information 3.

Further analysis was performed on 100 breeding lines from 3 crossing families (Table 3) by using primers hPb-719005 and hPb-CONT (Fig. 1). By using primers hPb-719005 and hPb-CONT the correct sex determination was achieved between 94% and 100% of cases, depending upon the marker used and the family, showing the importance of using all four male specific markers. Therefore, all 5 markers are required to maximise the likelihood that the assay is effective throughout the biogeographic range of hop. This is important as the large majority of hop in commercial production and breeding programs around the world is some form of multi-generation hybrid between European and North American germplasm.

### Development of multiplex PCR approach

Since neither hPb-CONT nor hPb-719005 were able to detect 100% known males independently, we aimed to develop a multiplex PCR approach where all four markers could be amplified simultaneously. In multiplex primer development the fifth sequence of hop chloroplast origin (contig18) was included to confirm amplification of isolated DNA and to exclude the risk of false negatives being identified as females. The developed multiplex primer set is presented in Table 2.

**Figure 2:**
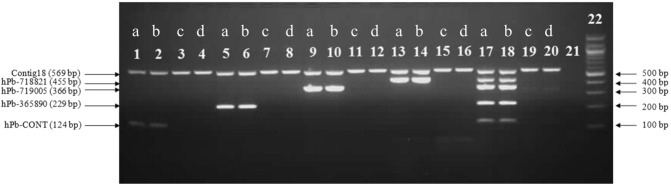
Verification of sex duplex and multiplex primer combinations. Samples a and b were male hop plants 2/1 and 3/3, while c and d female hop plants ‘Aurora’ and ‘Savinjski golding’. Lanes 1–4: PCR duplex hPb-CONT and contig18, lanes 5–8: PCR duplex hPb-365890 and contig18, lanes 9–12: PCR duplex hPb-719005 and contig18, lanes 13–16: PCR duplex hPb-718821 and contig18, lanes 17–20: multiplex reaction including all five markers hPb-CONT, hPb-365890, hPb-719005, hPb-718821 and contig18). Lane 21 is a negative control (DNA free) and lane 22 contains a 100 bp size marker. Full-length gel is presented in Supplementary Information 3.

During multiplex optimization the duplex PCR amplifications were performed by using non-sex specific chloroplast primer contig18 and four primers linked to male sex phenotype (hPb-CONT, hPb-719005, hPb-365890, hPb-718821 - Fig. 2).

**Figure 3:**
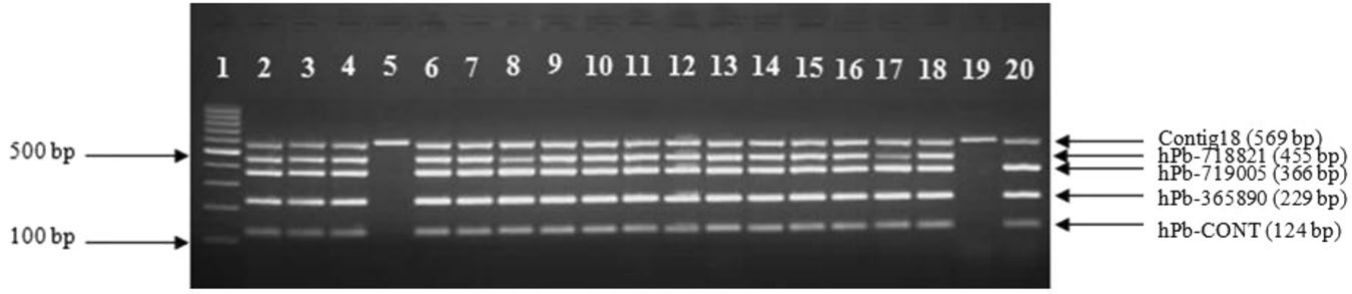
Multiplex PCR amplification with world hop accessions. Samples in lanes 5 and 19 are female, all others are male hop plants, lane 1 contains a 100 bp size marker. Genotypes analyzed are (lanes 2–20): 4/4, SLO5/2, SLO5/3, SLO1, SLO5/1, URATNIK, 21436, 13P10, 35PO1, JOŠT, 4OP15, 9/2, 2/137, SLO5/6, 2/1, 21426, 3/3, SLO2B/5 and 63012. Full-length gel is presented in Supplementary Information 3.

The verification of multiplex PCR amplification was initially checked on 97 genotypes (73 female and 24 male plants) representatives of globally significant hop accessions (Supplementary Information 1) by multiplying all five primers (hPb-CONT, hPb-365890, hPb-719005, hPb-718821, contig18). The markers successfully predicted sex phenotype in 97/97 cases (Fig. 3). In one male plant, a wild male genotype (63012 - Fig. 3, lane 20) of North American biogeographic origin, the primer hPb-718821 failed to amplify the fragment, even when the reaction was repeated. Nevertheless, the sex phenotype of the plant was correctly distinguished based on amplification of the other three male specific markers.

The results obtained from single PCR amplification and multiplex PCR amplification were compared. By using hPb-CONT and hPb-719005 primers, the results of sex determination in three crossing families coincided with phenotypic determination from 94% to 96% and 94% to 100%, respectively. Comparatively, when multiplex of five primers for PCR amplification was used in same progeny, 100% success was achieved. Furthermore, correct results were obtained by using multiplex PCR in the analyses of 96 world hop accessions (Supplementary Information 1).

### Direct PCR reaction using crude nucleic acid extract

**Figure 4:**
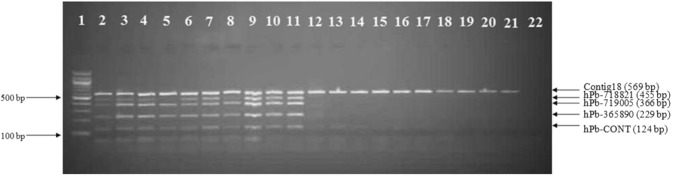
Crude sample multiplex PCR amplification. Samples in lanes 2–11 are male and in lanes 12–21 female hop plants, lane 22 is a negative control (DNA free) and lane 1 contains a 100 bp size marker. Genotypes analyzed are (lanes 2–21): 2/1, No3–38, 19058, 63012, 3/3, 273/110, 21426, 173/132, 41/39, 305/27, ‘Aurora’, ‘Magnum’, SLO2B/5, 81/54, ‘Styrian Fox’, ‘Styrian Dragon’, ‘Wye Target’, ‘Savinjski golding’, ‘Blisk’ and ‘Cicero’. Full-length gel is presented in Supplementary Information 3.

With the aim of accelerating sex determination in the hop breeding process, the possibility of using crude sample multiplex PCR amplifications was investigated on an initial 20 samples. In this initial test, the multiplexed markers correctly diagnosed the sex phenotype of each sample (Fig. 4).

Furthermore, in 14 families (a total of 253 genotypes – Table 4) the sex phenotype was diagnosed by crude sample multiplex PCR at seedling stage. After molecular analysis the plants were separately planted according to their marker determined sex genotype, in field trials within the context of the Slovenian hop breeding program. In the subsequent 1–2 years, phenotypic observations were collected and the comparison with molecular data was obtained. As can be seen from Table 4, the results from crude multiplex PCR amplification coincided perfectly with the in-field determination of plant sex phenotype.

## Discussion

In dioecious species, where plants of one sex phenotype are preferred for commercial use, breeding or cultivation, various DNA fingerprinting methods have been employed for sex determination, as described in species such as *Cannabis sativa*^39^, *Asparagus officinalis*^40,41^, *Actinidia chinensis*^42^, and *Ficus fulva*^43^. In some species such as *Garcinia gummi-gutta*^44^, *Simmondsia chinensis*^45^, and *Calamus guruba*^11^, the time between germination and the onset of reproductive maturity may be several years, imposing a strong limitation on the use of phenotypic determination of sex^9^. In hop, male plants cannot be differentiated phenotypically from female plants before flowering (sexual maturity), early sex determination via molecular markers is appealing. Beatson *et al*.^31^ reported the economic benefits of using a HRM sex marker for elimination of male seedlings at the nursery stage in several New Zealand triploid breeding populations.

The sex determination system is similar in *Humulus, Cannabis* and *Rumex* species, where the XX/XY system exists and the ratio between X chromosomes and autosomes effects sex expression^8,19^. In the family Cannabaceae, the three species – *Cannabis sativa, Humulus lupulus* and *Humulus japonicus* – are dioecious with heterogametic sex chromosomes. In *H. lupulus*, Ono^46^ described six different systems, where 1 to 3 pairs of sex chromosomes with different sizes of Y chromosome exist. Divashuk *et al*.^47^ reported a cytogenetic marker for the identification of sex chromosomes in *H. lupulus*. They revealed pseudo autosomal regions on the long arms of the X and Y chromosomes. Furthermore, Hill *et al*.^22^ identified a set of loci that are sex-linked and probably located in the pseudo autosomal region. Their study identified a 1.3 Mb section of DNA that appears unique to male hop genotypes. They proposed that the identified region could be utilised for the development of molecular markers for diagnosis of sex phenotype.

All reported sex determining molecular markers in hop, have been linked to the maleness. Polley *et al*.^6^ were the first to publish a sex-specific DNA sequence in hop, developed from random amplified polymorphic DNA (RAPD) molecular markers by using bulk segregant analysis (BSA), which is predominantly present on the Y chromosome and hybridized only weakly to female DNA. Upon testing^5,23^, this first sex-linked marker did not appear to coincide completely with the phenotypic assessments. Jakše *et al*.^28^ reported an SSR marker tightly linked to the male sex in hop, which appeared to show complete linkage to the male character but technical difficulties associated with SSR genotyping meant that this marker was not applied in marker assisted selection (MAS).

Hop breeding programs are long lasting, with a development timeline of 10 to 15 years from crossing to registration of a new cultivar. To improve the efficiency of traditional selection procedures, several different molecular markers have been used to analyse genetic distances among hop breeding genotypes^33,35,48–50^, and to detect QTLs (quantitative trait loci)^26–28,51,52^. Nevertheless, implementation of developed techniques in marker-assisted selection has remained unpublished up to this point. In order to avoid the delayed identification of male plants within the experimental systems of hop genetic improvement, we developed and applied selected DArT molecular markers to determine sex at an early hop seedling stage.

The present study was based on the research of Howard *et al*.^33^, where 730 polymorphic markers from 92 hop accessions were discovered using diversity arrays technology (DArT), which were further used in linkage studies^29^. There have been few studies describing the transferability of DArT markers identified through research, into breeding programs. In most cases markers were used in variability studies and genome mapping in different species, such as olive (*Olea europaea* L.)^53,54^, sugar beet (*Beta vulgaris* L.)^55^, apple (*Malus domestica* Borkh.)^56^, eucalypt (*Eucaplyptus* spp.)^57,58^, hexaploid wheat (*Triticum aestivum* L.)^59,60^, perennial ryegrass (*Lolium perenne* L.)^61^, and tomato (*Solanum lycopersicum* L.)^62^.

By studying sex linked marker performance in 197 plants (97 from the global hop genotype collection and 100 from crossing families), sex determined by multiplex PCR amplification appears to completely coincide with in-field phenotypic assessment. Furthermore, by using a crude sample multiplex PCR technique, the determination of sex in hop seedlings was made more efficient, without losing accuracy. The present research verified both single, and multiplex PCR sex-linked markers in 203 genotypes from world collection and three experimental families and developed and demonstrated the efficacy of a system of rapid, crude sample extract multiplex PCR sex-linked markers among progeny of 14 crosses.

To conclude, selected markers combined into a crude sample multiplex PCR assay, were tested in a broad spectrum of hop genetic resources, in progeny of crosses, and for routine application around 4.000 seedlings were annualy tested in the period of 2015–2018 in an active hop breeding program. It is important to note that taking into consideration all technical hours previously occupied in preventing male plants from flowering near commercial fields and the fact that accurate determination of sex at the seedling stage reduces the trial area required for screening by about one third, the financial cost of MAS has been recovered. Further, the laboratory analysis can be performed during winter, using leaf samples collected the previous growing season, spreading research activity away from seasonal labour peaks associated with hop production. The methods described above appear to produce complete linkage between multiplex PCR sex-linked molecular markers and phenotypic sex expression in field grown hop, and would appear to be appropriate for routine testing of hop seedlings in breeding programs worldwide.

## Supplementary information

Supplementary Information
