# Supplementary material for: New Male Specific Markers for Hop and Application in Breeding Program
Source: Sci Rep. 2019 Oct 2;9:14223. doi: 10.1038/s41598-019-50400-z (PMC6775077; doi:10.1038/s41598-019-50400-z)

**Andreja Čerenak^1*^, Zala Kolenc^1^, Petra Sehur^1^, Simon P Whittock^2^, Anthony Koutoulis^3^, Ron Beatson^4^, Emily Buck^4^, Branka Javornik^5^, Suzana Škof^5^, Jernej Jakše^5^**

**NEW MALE SPECIFIC MARKERS FOR HOP AND APPLICATION IN BREEDING PROGRAM**

^1^Slovenian Institute of Hop Research and Brewing, Cesta Žalskega tabora 2, 3310 Žalec, Slovenia

^2^Hop Products Australia, 446 Elizabeth Street, Hobart, Tasmania & School of Natural Sciences, University of Tasmania, Private Bag 55, Hobart, Tasmania

^3^University of Tasmania, School of Natural Sciences, Private Bag 55, Hobart TAS 7001, Australia

^4^The New Zealand Institute for Plant & Food Research Limited, Palmerston North Research Centre, Private Bag 11600, Palmerston North 4442, New Zealand
^5^University of Ljubljana, Biotechnical Faculty, Agronomy Department, Jamnikarjeva 101, 1000 Ljubljana, Slovenia

**Supplementary Information 1:** Hop material analyzed in presented study, material of crossing families is included in Table 3 and Table 4

Legend:

“-“ male marker not present

“+” male marker present

“(p)” length polymorphism of marker observed

“/” analysis not performed

**Cultivars and wild hops included in analysis (all females)**

| *Female hop* | *Origin* | *Cultivated/Wild* | hPb-CONT | hPb-719005 | hPb-365890 | hPb-718821 | *Multiplex* |
| --- | --- | --- | --- | --- | --- | --- | --- |
| Admiral | England | Cultivated | - | - | - | - | / |
| Agnus | Czech Republic | Cultivated | - | - | - | - | / |
| Ahil | Slovenia | Cultivated | - | - | - | - | / |
| Apolon | Slovenia | Cultivated | - | - | - | - | / |
| Aquila | USA | Cultivated | - | - | - | - | / |
| Aromat | Czech Republic | Cultivated | - | - | - | - | / |
| Atlas | Slovenia | Cultivated | - | - | - | - | / |
| Aurora | Slovenia | Cultivated | - | - | - | - | - |
| Apolon | Slovenia | Cultivated | / | / | / | / | - |
| Bačka | Serbia | Cultivated | - | - | - | - | / |
| Blisk | Slovenia | Cultivated | - | - | - | - | / |
| Bobek | Slovenia | Cultivated | - | - | - | - | - |
| Bor | Czech Republic | Cultivated | - | - | - | - | - |
| Braustern | Germany | Cultivated | - | - | - | - | / |
| Brewers Gold | England | Cultivated | - | - | - | - | - |
| Buket | Slovenia | Cultivated | - | - | - | - | - |
| Bullion | England | Cultivated | - | - | - | - | / |
| Calicross | New Zealand | Cultivated | - | - | - | - | / |
| Cascade | USA | Cultivated | - | - | - | - | - |
| Cekin | Slovenia | Cultivated | - | - | - | - | / |
| Celeia | Slovenia | Cultivated | - | - | - | - | - |
| Cerera | Slovenia | Cultivated | - | - | - | - | / |
| Chang Bei 1 | China | Cultivated | - | - | - | - | / |
| Chang Bei 2 | China | Cultivated | - | - | - | - | - |
| Chinook | USA | Cultivated | - | - | - | - | - |
| Cicero | Slovenia | Cultivated | - | - | - | - | / |
| Cluster | USA | Cultivated | - | - | - | - | - |
| Cobbs | England | Cultivated | - | - | - | - | - |
| Comet | USA | Cultivated | - | - | - | - | - |
| Crystal | USA | Cultivated | - | - | - | - | / |
| Dana | Slovenia | Cultivated | - | - | - | - | / |
| Diva | England | Cultivated | - | - | - | - | / |
| Dunav | Serbia | Cultivated | - | - | - | - | / |
| Early Bird Golding | England | Cultivated | - | - | - | - | - |
| El Lupulo | Argentina | Cultivated | - | - | - | - | - |
| Emerald | Germany | Cultivated | - | - | - | - | / |
| Estera | Poland | Cultivated | - | - | - | - | / |
| Eastwell golding | England | Cultivated | - | - | - | - | / |
| First Choice | New Zealand | Cultivated | - | - | - | - | - |
| Fuggle | England | Cultivated | - | - | - | - | - |
| Ging Dao Do Hua | China | Cultivated | - | - | - | - | - |
| Glacier | USA | Cultivated | - | - | - | - | - |
| Golden Star | USA | Cultivated | - | - | - | - | / |
| Groene Bel | Belgium | Cultivated | - | - | - | - | / |
| Hallertauer Gold | Germany | Cultivated | - | - | - | - | - |
| Hallertauer Mittelfrüher | Germany | Cultivated | - | - | - | - | - |
| Hallertauer Tradition | Germany | Cultivated | - | - | - | - | - |
| Harmony | Czech Republic | Cultivated | - | - | - | - | / |
| Herald | England | Cultivated | - | - | - | - | / |
| Hersbrucker | Germany | Cultivated | - | - | - | - | - |
| Hüller Aroma | Germany | Cultivated | - | - | - | - | / |
| Hüller Bitterer | Germany | Cultivated | - | - | - | - | - |
| Iwanovecki | Poland | Cultivated | - | - | - | - | / |
| Izabela | Poland | Cultivated | - | - | - | - | / |
| Kitamidori | Japan | Cultivated | - | - | - | - | - |
| Keyworth Midseason | England | Cultivated | - | - | - | - | - |
| Kirin 1 | Japan | Cultivated | - | - | - | - | / |
| Kostromsky | Russia | Cultivated | - | - | - | - | / |
| Kruglak siriak | Poland | Cultivated | - | - | - | - | / |
| Liberty | USA | Cultivated | - | - | - | - | - |
| Lubljinsky | Poland | Cultivated | - | - | - | - | / |
| Magnum | Germany | Cultivated | - | - | - | - | - |
| Mathon | England | Cultivated | - | - | - | - | / |
| Merkur | Germany | Cultivated | - | - | - | - | - |
| Nadwislandsky | Poland | Cultivated | - | - | - | - | / |
| Neoplanta | Serbia | Cultivated | - | - | - | - | / |
| Nordgard 1478 | Denmark | Cultivated | - | - | - | - | / |
| Nordgard 978 | Denmark | Cultivated | - | - | - | - | - |
| Northern Brewer | England | Cultivated | - | - | - | - | - |
| Nugget | USA | Cultivated | - | - | - | - | - |
| Omega | USA | Cultivated | - | - | - | - | / |
| Osvaldov klon 126 | Czech Republic | Cultivated | - | - | - | - | / |
| Osvaldov klon 72 | Czech Republic | Cultivated | - | - | - | - | - |
| Outeniqua | South African Republic | Cultivated | - | - | - | - | / |
| Pacific Gem | New Zealand | Cultivated | - | - | - | - | / |
| Perle | Germany | Cultivated | - | - | - | - | - |
| Petrovački červenjak | Czech Republic | Cultivated | - | - | - | - | / |
| Poljski klon 12 | Poland | Cultivated | - | - | - | - | / |
| Precoce de Bourgogne | France | Cultivated | - | - | - | - | / |
| Premiant | Czech Republic | Cultivated | - | - | - | - | / |
| Pride of Kent | England | Cultivated | - | - | - | - | / |
| Pride of Ringwood | Australia | Cultivated | - | - | - | - | / |
| Record | Belgium | Cultivated | - | - | - | - | / |
| Ringwood Special | Australia | Cultivated | - | - | - | - | / |
| Saazer | Czech Republic | Cultivated | - | - | - | - | - |
| Saladin | Germany | Cultivated | - | - | - | - | / |
| Sara | Belgium | Cultivated | - | - | - | - | / |
| Savinjski golding | Slovenia | Cultivated | - | - | - | - | - |
| Serebrjanka | Russia | Cultivated | - | - | - | - | - |
| Sirem | Czech Republic | Cultivated | - | - | - | - | / |
| Smooth Cone | New Zealand | Cultivated | - | - | - | - | / |
| Southern Brewer | South African Republic | Cultivated | - | - | - | - | - |
| Southern Star | South African Republic | Cultivated | - | - | - | - | / |
| Spalter | Germany | Cultivated | - | - | - | - | / |
| Sterling | USA | Cultivated | - | - | - | - | / |
| Strisselspat | France | Cultivated | - | - | - | - | - |
| Symphony | USA | Cultivated | - | - | - | - | / |
| Tardif de Bourgone | France | Cultivated | - | - | - | - | / |
| Tettnanger | Germany | Cultivated | - | - | - | - | - |
| Topaz | Australia | Cultivated | - | - | - | - | / |
| Tutsham | England | Cultivated | - | - | - | - | - |
| Univerzal | Russia | Cultivated | - | - | - | - | / |
| Urožajni | Russia | Cultivated | - | - | - | - | - |
| White Bine | Belgium | Cultivated | - | - | - | - | / |
| Wye Nortdown | England | Cultivated | - | - | - | - | - |
| Würtemberger | Germany | Cultivated | - | - | - | - | / |
| Wye Challenger | England | Cultivated | - | - | - | - | - |
| Wye Saxon | England | Cultivated | - | - | - | - | / |
| Wye Target | England | Cultivated | - | - | - | - | - |
| Wye Viking | England | Cultivated | - | - | - | - | / |
| Yeoman | England | Cultivated | - | - | - | - | - |
| Zenith | England | Cultivated | - | - | - | - | - |
| Zlatan | Czech Republic | Cultivated | - | - | - | - | / |
| Žateški polurani červenjak | Czech Republic | Cultivated | - | - | - | - | / |
| Žitomirski klon 18 | Ukraine | Cultivated | - | - | - | - | / |
| Žitomirski klon 34 | Ukraine | Cultivated | - | - | - | - | / |
| Wild Germany Bavaria | Germany | Wild | - | - | - | - | / |
| Wild Austria | Austria | Wild | - | - | - | - | / |
| Wild Germany Berlin | Germany | Wild | - | - | - | - | / |
| Wild Italy | Italy | Wild | - | - | - | - | / |
| 1441.001 (var. neomexicana) | USA | Wild | - | - | - | - | / |
| 14AHJ | Bosnia and Herzegovina | Wild | / | / | / | / | - |
| 15AHJ | Serbia | Wild | / | / | / | / | - |
| 1AHJ | Croatia | Wild | / | / | / | / | - |
| 20AHJ | Serbia | Wild | / | / | / | / | - |
| 21AHJ | Serbia | Wild | / | / | / | / | - |
| 2AHJ | Croatia | Wild | / | / | / | / | - |
| 3AHJ | Croatia | Wild | / | / | / | / | - |
| 6AHJ | Croatia | Wild | / | / | / | / | - |
| 9AHJ | Bosnia and Herzegovina | Wild | / | / | / | / | - |
| A12 | Russia (Altai) | Wild | / | / | / | / | - |
| A16 | Russia (Altai) | Wild | / | / | / | / | - |
| AH17 | Slovenia | Wild | / | / | / | / | - |
| AH19 | Slovenia | Wild | / | / | / | / | - |
| AH22 | Slovenia | Wild | / | / | / | / | - |
| AH25 | Slovenia | Wild | / | / | / | / | - |
| AH7 | Slovenia | Wild | / | / | / | / | - |
| AH9 | Slovenia | Wild | / | / | / | / | - |
| K11 | Georgia (Kaucasus) | Wild | / | / | / | / | - |
| K11 | Georgia (Kaucasus) | Wild | / | / | / | / | - |
| K5 | Georgia (Kaucasus) | Wild | / | / | / | / | - |
| R15 | Russia | Wild | / | / | / | / | - |
| R15 | Russia | Wild | / | / | / | / | - |
| R24 | Russia | Wild | / | / | / | / | - |
| R29 | Russia | Wild | / | / | / | / | - |
| R32 | Russia | Wild | / | / | / | / | - |
| R41 | Russia | Wild | / | / | / | / | - |

**Male hops included in analysis**

| *Male hop* | *Origin* | *Cultivated/Wild* | hPb-CONT | hPb-719005 | hPb-365890 | hPb-718821 | *Multiplex* |
| --- | --- | --- | --- | --- | --- | --- | --- |
| 2/1 | Slovenia | Wild | + | + | + | + | + |
| 3/3 | Slovenia | Wild | + | + | + | + | + |
| 19058 | USA | Wild | +(p) | - | - | - | + |
| 29-70-54 | Great Britain | Wild | + | + | + | + | / |
| 64035 | USA | Wild | + | + | + | + | / |
| No3-38 | Japan | Wild | - | - | + | + | + |
| 305/27 | USA/EU | Wild | + | + | + | + | / |
| 2/137 x=40 | Slovenia | Wild | + | + | + | + | + |
| 310/48 | USA/EU | Wild | + | + | + | + | / |
| 300/166 | USA/EU | Wild | + | + | + | + | / |
| 24/80 | USA/EU | Wild | + | + | + | + | / |
| 306/104 | USA/EU | Wild | + | + | + | + | / |
| 85/169 | USA/EU | Wild | +(p) | - | + | + | / |
| 120/13 | USA/EU | Wild | + | - | - | + | / |
| 306/52 | USA/EU | Wild | + | + | + | + | / |
| 162/75 | EU/USA | Wild | + | + | + | + | / |
| 21340 | USA | Wild | + | + | + | + | + |
| 305/28 | USA/EU | Wild | + | + | + | + | / |
| 306/70 | USA/EU | Wild | + | + | + | + | / |
| 284/113 | USA/EU | Wild | - | + | + | + | / |
| 244/27 | USA/EU | Wild | +(p) | + | + | + | / |
| 241/150 | USA/EU | Wild | +(p) | + | + | + | / |
| 233/81 | EU | Wild | + | + | + | + | / |
| 227/69 | EU | Wild | + | + | + | + | / |
| 227/108 | EU | Wild | + | + | + | + | / |
| 3012 | EU | Wild | + | - | - | - | / |
| 21436 | USA | Wild | +(p) | - | + | - | + |
| 21426 | USA | Wild | + | - | - | - | + |
| OB21 | United Kingdom | seedling of Brewers Gold | + | - | + | + | / |
| INT101 | Japan | Wild | - | - | + | - | + |
| 1367.001 | USA | Wild | +(p) | - | + | - | / |
| 1401.001 | USA | Wild | +(p) | - | + | - | / |
| 20P09 | Croatia | Wild | + | + | + | + | + |
| 4/4 | Slovenia | Wild | + | + | + | + | + |
| SLO 5/2 | Slovenia | Wild | + | + | + | + | + |
| SLO 5/3 | Slovenia | Wild | + | + | + | + | + |
| SLO 5/1 | Slovenia | Wild | + | + | + | + | + |
| URATNIK | Slovenia | Wild | + | + | + | + | + |
| 13P10 | Croatia | Wild | + | + | + | + | + |
| 35P01 | Croatia | Wild | + | + | + | + |  |
| JOŠT | Slovenia | Wild | + | + | + | + | + |
| 4OP15 | FRY Macedonia | Wild | + | + | + | + | + |
| 9/2 | Slovenia | Wild | + | + | + | + | + |
| Wild Slovenia | Slovenia | Wild | + | - | + | + |  |
| 63012 | USA | Wild | / | / | / | / | + |
| 27P17 | Slovenia | Wild | / | / | / | / | + |
| 31/107 | Slovenia | Wild | / | / | / | / | + |
| 07P14 | Slovenia | Wild | / | / | / | / | + |
| 35P01 | Serbia and Montenegro | Wild | / | / | / | / | + |

**Monoecious hops included in analysis**

Legend:

Mf: predominatly male plants

Fm: predominatly female plants

FM: equal share of male and female flowers

| *Monoecious hop* | *Phenotype* | hPb-CONT | hPb-719005 | hPb-365890 | hPb-718821 | *Multiplex* |
| --- | --- | --- | --- | --- | --- | --- |
| 131/94 | Mf | + | + | + | + | / |
| 131/98 | Mf | + | - | + | + | / |
| 131/113 | Mf | + | - | - | + | / |
| 132/24 | Fm | - | - | - | - | / |
| 132/39 | Fm | - | - | - | - | / |
| 132/45 | Fm | - | - | - | - | / |
| 132/79 | Mf | + | + | + | + | / |
| 51/182 | Mf | + | - | + | + | / |
| 58/55 | Mf | + | - | + |  | / |
| 50/196 | Mf | - | - | - | + | / |
| 71/70 | Mf | + | - | + | + | / |
| 71/158 | Mf | + | + | + | + | / |
| 152/90 | Mf | + | + | + | + | / |
| 161/100 | Mf | + | - | + | + | / |
| 160/80 | Mf | + | + | + | + | / |
| 160/95 | Mf | + | - | + | + | / |
| 158/51 | Fm | - | - | - | - | / |
| 158/68 | Fm | - | - | - | - | / |
| 159/24 | Mf | + | + | + | + | / |
| 131/59 | Fm | - | - | - | - | / |
| 131/73 | Fm | - | - | - | - | / |
| 131/74 | Fm | - | - | - | - | / |
| 131/85 | Fm | - | - | - | - | / |
| 132/13 | Fm | + | - | + | - | / |
| 132/71 | Fm | - | - | - | - | / |
| 132/80 | Fm | - | - | - | - | / |
| 132/73 | Fm | - | - | - | - | / |
| 131/76 | Fm | - | - | - | - | / |
| 161/55 | Mf | + | + | + | + | / |
| 174/34 | Mf | + | + | + | + | / |
| 174/37 | Mf | + | + | + | + | / |
| 174/45 | Mf | + | - | + | + | / |
| 174/49 | Mf | + | - | + | + | / |
| 188/156 | Fm | - | - | - | - | / |
| 190/21 | Fm | - | - | - | - | / |
| 186/237 | Mf | + | + | + | - | / |
| 187/95 | FM | - | - | - | - | / |

**Supplementary Information 2**: Sequence alignment of DArT markers hPb-718886, hPb-363461 and hPb-361327

hPb-718886 TGCAGGGTGTGCAAGGTTGAAATTCACAGTCTGCCTGTTTGATGCTATTAAGGAAAGTTT

hPb-363461 TGCAGGGTGTGCAAGGTTGAAATTCACAGTCTGCCTGTTTGATGCTATTAAGGAAAGTTT

hPb-361327 TGCAGGGTGTGCAAGGTTGAAATTCACAGTCTGCCTGTTTGATGCTATTAAGGAAAGTTT

************************************************************

hPb-718886 TAAGTTGAA**A**GATTTTAGGGCCATTATTTAGATTGAATCTTTCATGAAGATCGGTCC**A**CA

hPb-363461 TAAGTTGAA**A**GATTTTAGGGCCATTATTTAGATTGAATCTTTCATGAAGATCGGTCC**A**CA

hPb-361327 TAAGTTGAA**G**GATTTTAGGGCCATTATTTAGATTGAATCTTTCATGAAGATCGGTCC**G**CA

********* *********************************************** **

hPb-718886 TATCCATAGCTTGATCTTGAAACATGATACTAGAGGCAATTCAATTAGAAACAAATTGGA

hPb-363461 TATCCATAGCTTGATCTTGAAACATGATACTAGAGGCAATTCAATTAGAAACAAATTGGA

hPb-361327 TATCCATAGCTTGATCTTGAAACATGATACTAGAGGCAATTCAATTAGAAACAAATTGGA

************************************************************

hPb-718886 GAGTCCAAGCCATGGCCATATTATTGCATTGAATCCATGAAGAATAATTGGGATCATCAG

hPb-363461 GAGTCCAAGCCATGGCCATATTATTGCATTGAATCCATGAAGAATAATTGGGATCATCAG

hPb-361327 GAGTCCAAGCCATGGCCATATTATTGCATTGAATCCATGAAGAATAATTGGGATCATCAG

************************************************************

hPb-718886 C**G**GGTGGGTCAGGCAGAGGGCCATCGATGAACGGAGTCTTGTTCTTGGCGCCATTAGAGA

hPb-363461 C**A**GGTGGGTCAGGCAGAGGGCCATCGATGAACGGAGTCTTGTTCTTGGCGCCATTAGAGA

hPb-361327 C**A**GGTGGGTCAGGCAGAGGGCCATCGATGAACGGAGTCTTGTTCTTGGCGCCATTAGAGA

* **********************************************************

hPb-718886 TCGATGCGGCTCGTTTCTAGAACTAAAAATTTTCTTCGCCTGTGAGAGAAGTGCGGACAA

hPb-363461 TCGATGCGGCTCGTTTCTAGAACTAAAAATTTTCTTCGCCTGTGAGAGAAGTGCGGACAA

hPb-361327 TCGATGCGGCTCGTTTCTAGAACTAAAAATTTTCTTCGCCTGTGAGAGAAGTGCGGACAA

************************************************************

hPb-718886 GGAGATAATTGGGATTATCTTCGGTGCTCTAATGGTAGGGACTAGTGCAGTCCTCGTGAG

hPb-363461 GGAGATAATTGGGATTATCTTCGGTGCTCTAATGGTAGGGACTAGTGCAGTCCTCGTGAG

hPb-361327 GGAGATAATTGGGATTATCTTCGGTGCTCTAATGGTAGGGACTAGTGCAGTCCTCGTGAG

************************************************************

hPb-718886 CAAGTTGATCACGAGGTTCGATTGGAGTGAAGCGATTTGCTGCA

hPb-363461 CAAGTTGATCACGAGGTTCGATTGGAGTGAAGCGATTTGCTGCA

hPb-361327 CAAGTTGATCACGAGGTTCGATTGGAGTGAAGCGATTTGCTGCA

********************************************

**Supplementary Information 3:** Full-length gels of Figures 1 – 4. Red squares are marking cropped parts of gels presented in the Manuscript.

Figure 1:


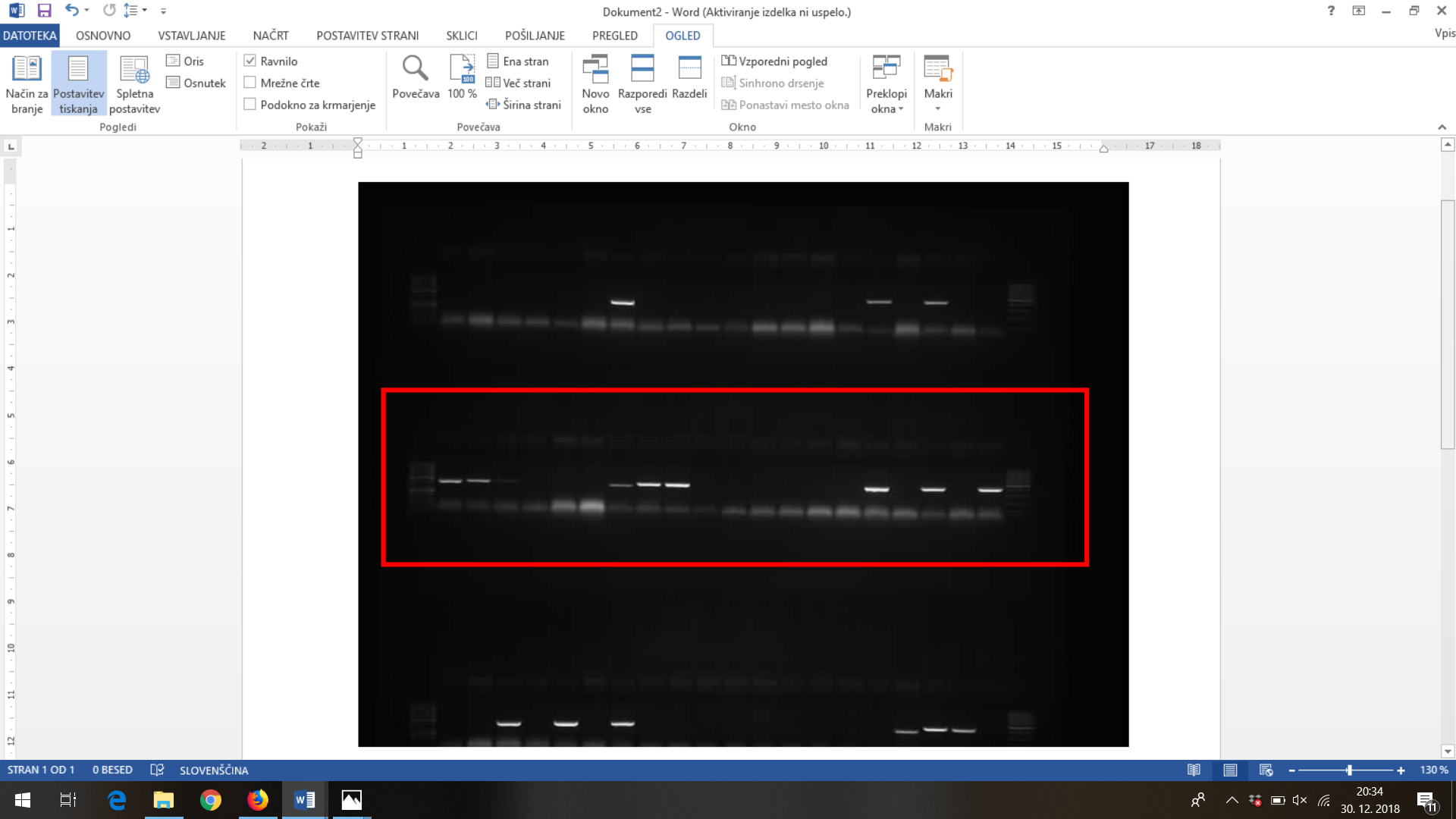


Figure 2:


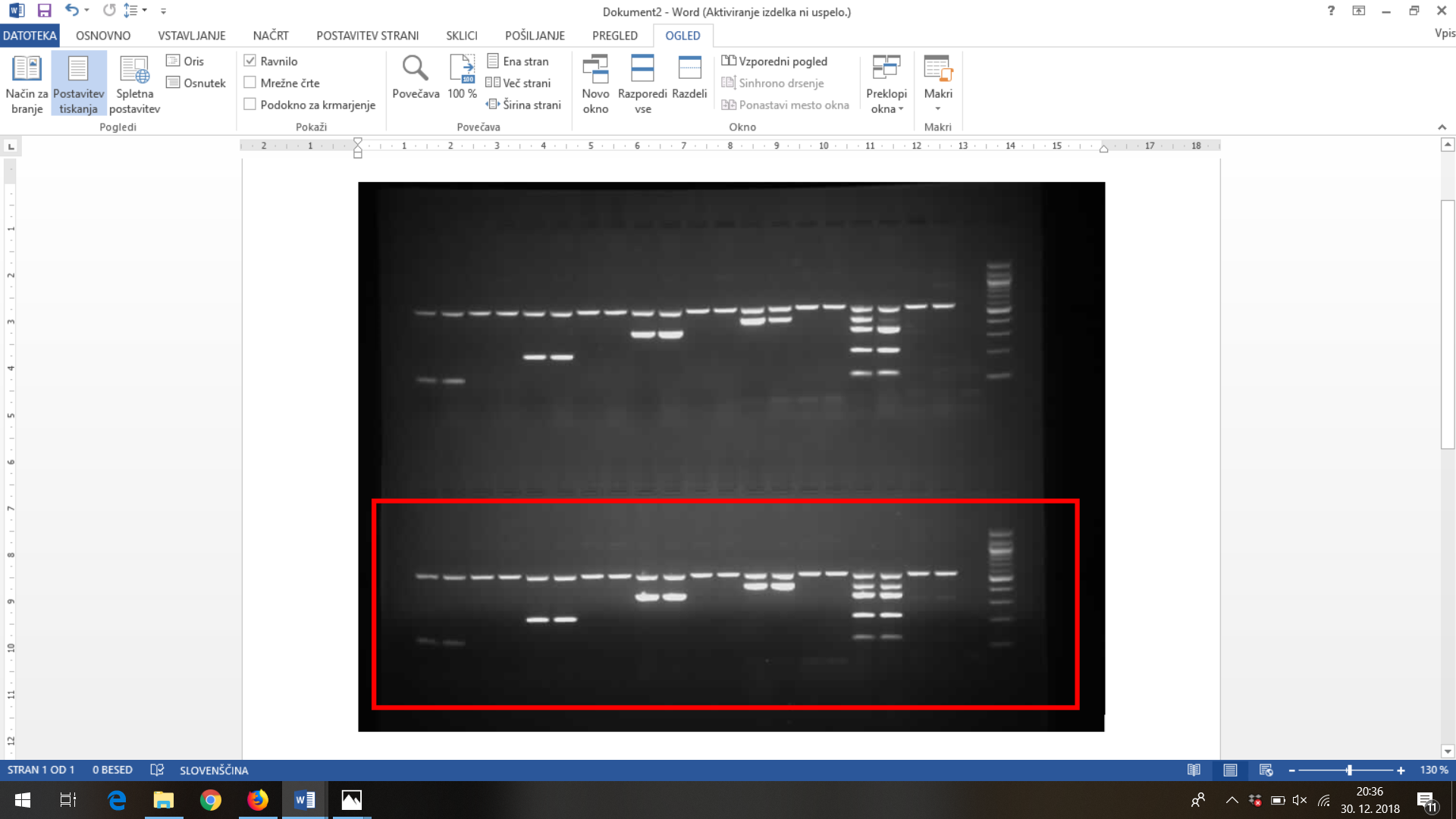


Figure 3:


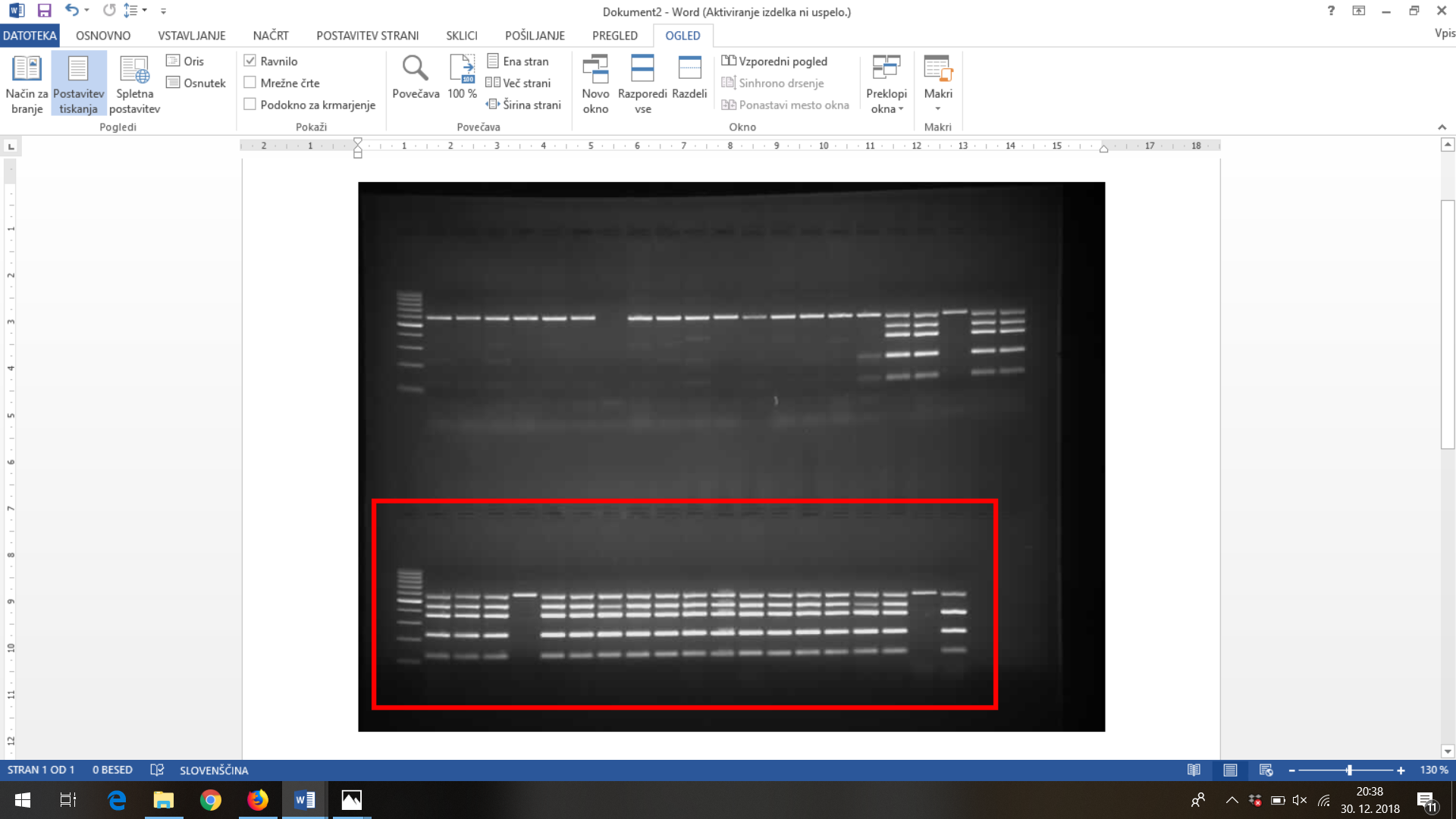


Figure 4:


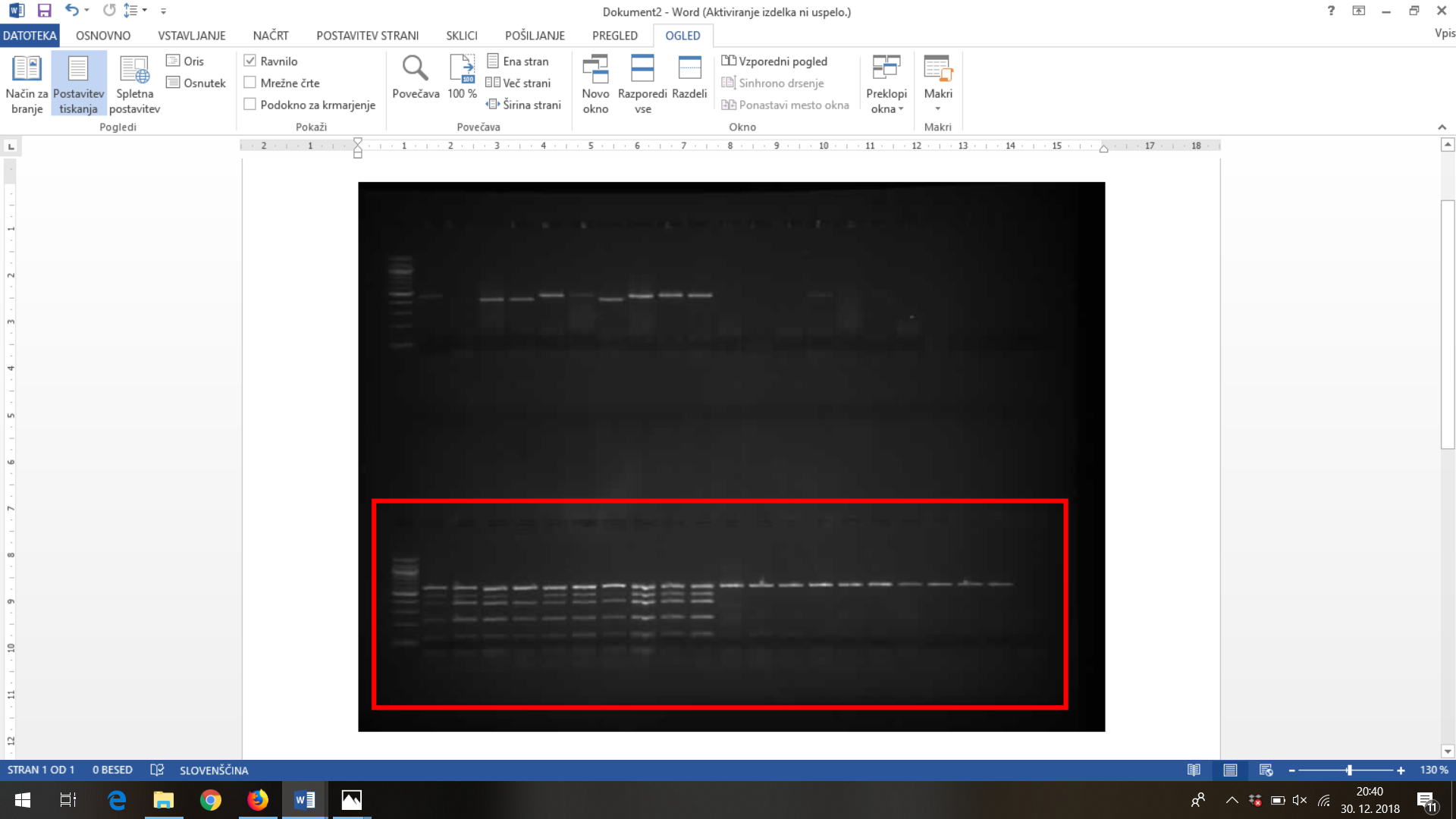

Supplement: Supplementary file 1 — Supplementary Information [file 41598_2019_50400_MOESM1_ESM.docx]
